# Merits and Limitations of Robotic-assisted Surgery in Improving Precision, Accuracy, and Patient Outcomes in Orthopedic Procedures

**DOI:** 10.26502/josm.511500243

**Published:** 2025-12-15

**Authors:** David Parvizi, Blake Han, Artin Allahverdian, Devendra K Agrawal

**Affiliations:** 1 Department of Translational Research, College of Osteopathic Medicine of the Pacific, Western University of Health Sciences, Pomona, California, USA; 2 California University of Science and Medicine, Colton, California, USA

**Keywords:** Clinical and functional outcomes, Cost-effectiveness and workflow efficiency, Intraoperative navigation, Learning curve and surgeon performance, Minimally invasive techniques, Patient outcomes, Robotic-assisted orthopedic surgery, Spinal fusion robotics, Surgical precision and accuracy, Total hip arthroplasty, Total joint arthroplasty, Total knee arthroplasty

## Abstract

Since the 1980s, research in robotic-assisted orthopedic surgery has shown to improve precision and accuracy in implant positioning and alignment, specifically for knee and hip arthroplasty. The current meta-analyses and randomized controlled trials have shown that robotic systems are able to consistently reduce alignment outliers and lead to improved radiological outcomes. Some evidence has also concluded small, yet significant, improvements for short-term, patient-reported postoperative pain. Although modest improvements have been demonstrated in the shortterm, long-term technical improvements have not been established. Patient satisfaction, functional outcomes, and rates of revision and complications are still under investigation in terms of long-term outcomes. Moreover, clinical impact is also variable between different robotic platforms, surgeon experience, and healthcare setting. High costs, steep learning curves, and increased operative costs are major limitations to the implementation of widespread use of robotic systems. Altogether, robotic-assisted orthopedic surgery may improve technical accuracy and precision, however, its limitations such as inconsistent clinical benefit, increased operating time, and high cost begs the question whether these systems will be implemented in hospital systems. Further long-term studies must be done to conclude whether robotic assistance plays a significant role in improving clinical outcomes.

## Introduction

Over the last two decades, robotic-assisted surgery has expanded its reach in orthopedic surgery, with the first systems emerging in the 1980s and its first implementation in the 1990s which focused on arthroplasty and spinal instrumentation. ROBODOC was one of the first systems about robotic orthopedic surgery and was designed to improve the precision of femoral canal preparation in total hip arthroplasty (THA) [1]. Over the last twenty years, newer robotic systems have transitioned to semi-active modalities which provide haptic feedback allowing surgeons to execute preoperative plans with improved consistency and control. The MAKO system, which is now widely used in hip and knee arthroplasty (TKA), emphasizes the transition to platforms that integrate 3D imaging, real-time navigation, and surgeon-guided robotic arms [2]. The MAKO robotic-assisted surgical system can be used by surgeons to aid in precision and accuracy in procedures such as TKA and THA. The system works by first using a preoperative CT scan to create a 3D model of the patient allowing for detailed planning of the size of the implant, position, and alignment of surgical instruments all before beginning surgery [3]. The MAKO system has shown improved accuracy and precision of component position in comparison to manual techniques, with a large proportion of implants being within two degrees of the targeted alignment [4]. The accuracy that the MAKO system achieves reduces misalignment which decreases the risk of implant malposition. This system also can aid in intraoperative gap balancing and soft tissue assessment, further improving individualized adjustments to optimize joint mechanics [5].

The three classified systems for robotics in orthopedics are as follows: autonomous, semi-autonomous, or teleoperated which are further differentiated between open versus closed platforms as well as image-based and imageless navigation. Implant positioning, bone preparation, and alignment are the three aspects that have shown the most improvement using robotics. Radiologic studies have consistently shown reduced variability and reproducibility with robotic assistance compared to manual surgical techniques [6]. The key determinants of biomechanical function and implant longevity are directly related to implant positioning, alignment, and bone resections which is why precision and accuracy are so important. Robotic systems have consistently demonstrated less deviation from planned alignment and component positioning compared to manual instruments resulting in a larger proportion of implants placed with optimal parameters [7]. By having improved precision and accuracy of implant placement via robotic assisted surgery, facilitation of joint kinematics can be achieved more efficiently leading to less postoperative pain, improved early functional outcomes, and enhanced patient satisfaction [8]. Although long-term data outcomes are still emerging, the reduction in technical variability and outliers is considered an important precondition for improved implant survivorship and decreased rates of revision. Altogether, precision and accuracy play a crucial role as they underpin the technical success of orthopedic procedures and are associated with improved short-term outcomes and can possibly contribute to improved long-term results.

Current patient expectations for robotic-assisted orthopedic surgery are that it provides superior precision and accuracy in implant positioning and alignment, however, the literature does not consistently demonstrate superior patient-reported outcomes or long-term functional results [9]. Robotic-assisted total hip and total knee arthroplasty achieve higher rates of accurate component placement, reduce leg-length discrepancies, and can lower complication rates including periprosthetic infection and mechanical complications [10]. Multiple systematic reviews, metaanalyses, and randomized controlled trials have shown that patient-reported outcomes of robotic vs. manual surgical techniques, based on scales such as the Harris Hip Score and Western Ontario and McMaster Universities Osteoarthritis Index, have demonstrated minimal clinical difference at short to mid-term follow up [11,12]. Moreover, it has been shown that rates of revision and implant longevity are comparable between the two techniques. Robotic-assisted systems require longer operative times and higher costs but can offset this by offering reduced length of stay and lower rates of perioperative complications. In summary, robotic-assisted surgery has shown to improve precision and accuracy as well as reduce some complications, however, current evidence does not show a consistent or clinically significant improvement in overall patient outcomes compared to manual surgical practices.

The main objective of this paper is to critically evaluate the available evidence about the advantages and disadvantages of robotic-assisted orthopedic surgery. Specifically, this review will analyze in terms of precision, accuracy, and patient outcomes as well as address the implications of these findings for future clinical practice and research. This article synthesized data from systematic reviews, meta-analyses, and clinical studies to evaluate whether robotic systems can consistently improve technical metrics such as implant positioning and alignment, and whether these advancements would be able to translate into functional outcomes, satisfaction, and complication rates. By evaluating the advantages, such as enhanced reproducibility and reduced technical variability, as well as the disadvantages including increased operative time, cost, and disputable long-term outcomes, this analysis will aim to inform clinicians and researchers about the current state of robotic-assisted orthopedic surgery and pinpoint areas where further research is needed to guide optimal integration into modern surgical practice.

## Methods

The PubMed, Embase, and Cochrane databases were searched using the terms “orthopedic surgery,” “orthopedic-assisted robotic surgery,” “prosthesis,” “image-guided orthopedic surgery,” “surgical accuracy in orthopedics,” and “MAKO robotic system.” Any randomized controlled trial, cohort study, or systematic review published in English during the period of 2000 to 2025 was included. Studies were not limited to either pediatric or adult populations. Titles and abstracts were screened for relevance by the authors, and full texts were reviewed for final inclusion. Case reports, abstracts, and the level of evidence was also noted based on study design. Studies were categorized by intervention aimed at assessing the usability of robotic surgery in orthopedics. Primary outcomes were surgical accuracy and precision, functional recovery, and health-related quality of life.

## Merits of Robotic-Assisted Surgery

### Precision and Accuracy

**Figure 1:**
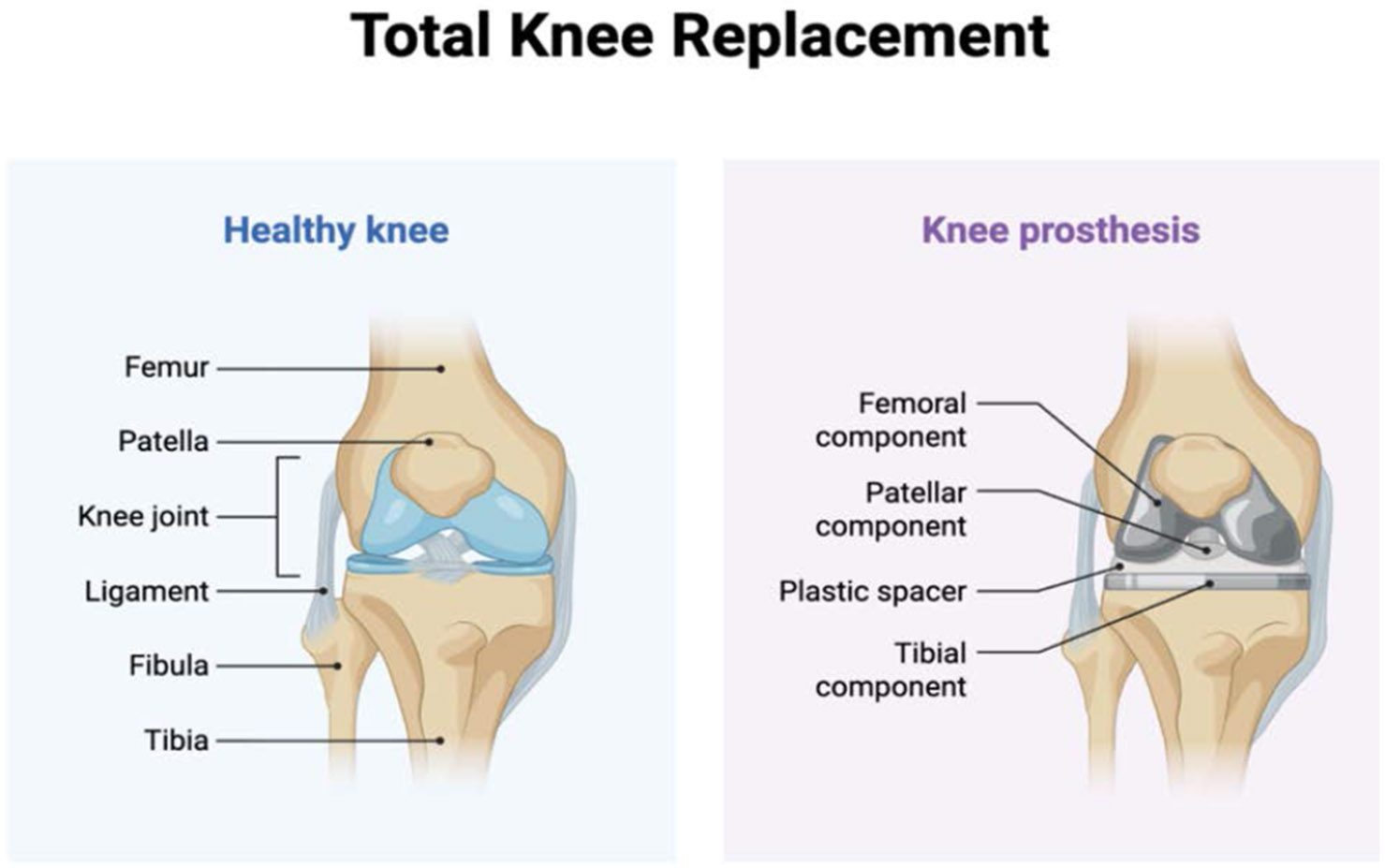
This image compares a healthy knee joint with a total knee replacement, showing how natural structures like cartilage and ligaments are placed with prosthetic components including femoral, tibial, patellar and plastic spacer implants. Robotic-assisted surgery enhances the precision of placing these components, improving aligment, reducing variability and optimizing patient outcomes.

Navigation techniques and robotic-assisted systems have led to significant improvements in positioning during orthopedic surgery through the integration of preoperative imaging, real-time intraoperative tracking, and computer-guided execution (Figure 1). This process starts with a high-resolution MRI or CT scan which is used to generate a three-dimensional model of the patient's anatomy. Optical or electromagnetic tracking systems are then used during surgery to monitor the positioning of surgical instruments and implants in real time. Through these techniques, surgeons can detail their intraoperative planning through proper selection of implant size, orientation, and alignment targets [13]. Moreover, navigation platforms can provide visual feedback directly to the surgeon through displaying instrument trajectories and implant positions relative to the planned target. Semi-active platforms can also add haptic boundaries and robotic arm guidance to constrain bone resections and implant placement within a small margin of error to significantly reduce human error and reduce technical variability [14]. These systems are most often used in TKA, UKA, THA, shoulder arthroplasty, and spine surgery. The use of robotics has shown improvement by facilitating accurate pedicle screw placement and interbody fusion, further reducing the risk of neurovascular injury [15]. Regarding joint arthroplasty, robotic systems optimize component alignment and gap balancing, which are essential for the restoration of insufficiency biomechanics [16]. This process is used in every step of surgery, with preoperative planning and imaging, intraoperative registration of patient-specific anatomy, real-time instrument tracking, and computer-guided or robotic-assisted execution of bone cuts and implant placement. The advantage is that the surgeon remains in complete control of decision-making, with the system only enhancing precision and reproducibility [17].

The literature supports that robotic-assisted surgery improves implant positioning, precision, and accuracy compared to manual surgical techniques. Meta-analyses and randomized controlled trials have demonstrated that robot-assisted THA and TKA resulted in a larger proportion of implants placed within established safe zones, reduced limb length discrepancy, and higher accuracy of biomechanical restoration [18]. Robotic THA can achieve more accurate cup placement and increased control of limb length and hip offset, while robotic TKA and UKA show improved component alignment by an average of 2-3 mm and reduced outliers in the mechanical axis [19]. However, the data indicated that functional scores, satisfaction, and revision rates are generally equivalent between robotic-assisted and manual techniques at short and mid-term follow up, despite the technical improvements associated with robotic-assisted surgery [20]. Patient reported outcomes such as Harris Hip Score and Western Ontario and McMaster Universities Arthritis index are equivalent between robotic and manual techniques at short and long-term. Several studies have reported modest improvements in early pain and range of motion; however, these do not consistently translate into clinically meaningful differences or increased rates of attaining clinically significant improvements [21]. The long-term data is limited and does not clearly indicate that robotic-assisted orthopedic surgery is superior to manual techniques [22]. Furthermore, rates of complication in robot-assisted vs. manual surgery is 0.5% versus 3.1%, specifically complications including misalignment, mechanical errors, and similar 90-day dislocation rates, however, no study has shown a consistent reduction in complications or revision rates [23]. The disadvantage that many physicians face is that robotic surgery is associated with longer operative times, higher initial costs, and a steep learning curve for learning how to use the instruments [24]. Costs for robotic-assisted procedures are also much higher compared to manual conventional surgery. Robotic TKA has a median totla cost per case of $11,615 compared to $8,674 for a manual TKA [25]. Moreover, the facility cost associated with robotic surgery is 1.08-1.19 times higher.

### Patient Outcomes

Functional outcomes including pain scores and range of motion are shown to me comparable between robotic-assisted and manual orthopedic techniques (Figure 1). The data shows that for robot-assisted UKA there have been improved pain scores, decreased opioid requirements, and increased maximal knee flexion at discharge (mean 110.6° vs. 101.5°, p <0.001) compared to manual UKA [26]. Robotic-assisted TKA has also shown similar results, with slightly lower pain scores during movement at 24 hours post op, reduced opioid use in the first 2 days post-op, and shorter duration of stay in the hospital compared to manual TKA [27]. Range of motion is similar at discharge, but some studies have demonstrated that manual TKA is able to obtain greater maximal flexion at 1 year, 120.3° vs. 117.8° for robot-assisted [28]. The Western Ontario and McMaster Universities Osteoarthritis Index (WOMAC), Knee Society Score (KSS), and Oxford Knee Score (OKS) have been shown to be largely equivalent across multiple studies between robotic and manual techniques at one to five years [29]. Several small studies have shown minimal improvement in pain and function scores for robotic-assisted TKA at one to two years with mean WOMAC pain at 1 + 2 vs. 2 + 3 for manual techniques [30].

Robotic surgery has shown a decreased rate of complications, particularly in joint arthroplasty. A large, multi-center study of THA showed that 90-day readmission rates for robotic-assisted THA was significantly lower compared to those who underwent manual THA, as well as fewer readmissions for dislocation postoperatively. Moreover, surgical site infection and cellulitis rates were both significantly decreased in the robotic-assisted THA groups. Lastly, no significant difference was found in emergency department visits, however, robot-assisted THA had an increased number of visits for dyspnea without pulmonary embolism [31]. In terms of TKA, robotic-assisted surgery had less revisits and readmissions with >23 hours of observation. Robot-assisted TKA also was associated with less revisits for joint stiffness, chronic pain, and acute injuries [32]. For UKA, meta-analysis showed robotic-assisted UKA has fewer complications and lower revision rates compared to conventional measures [33]. In terms of spine surgery, the most improvement for robotic-assisted procedures is in lumbar fusions. It is associated with lower rates of heart failure, acute coronary artery disease, pulmonary edema, venous thromboembolism, and traumatic spinal injury; however, it has higher rates of post-surgical anemia, blood transfusion, and acute kidney injury compared to manual techniques [34]. Other studies have suggested that there is no significant difference in 90-day rates of wound complications, infection, or readmission in robotic vs. manual [35]. Complications for manual techniques generally include dislocation, surgical site infection, joint stiffness, hematoma, and mechanical complications. Unique risks that are found in robotic-assisted surgeries include pin-hole fractures, pin-related infection, iatrogenic soft tissue injury, and technical issues including retained registration checkpoints or unexpected robotic arm movement; however, these are rare and usually related to system-specific factors [36].

Altogether, robotic-assisted orthopedic surgery can provide modest early improvements in pain and range of motion, however, the long-term functional outcomes are like manual surgical techniques. There is no direct benefit over using robotic vs. manual techniques in terms of functional outcomes. Robotic techniques have also shown to reduce certain complication rates such as early dislocation and infection but also introduce few unique but infrequent technology-related risks.

## Limitations of Robotic-Assisted Surgery

Despite the advantages of robotic assistance in orthopedic procedures, there are several limitations to its widespread implementation. Technical issues are common, with system errors and malfunctions requiring intraoperative troubleshooting and leading to extended operative times. A large 14-year retrospective analysis found that system errors contributed to 7.4% of adverse events associated with robotic procedures. These errors were responsible for 82% of system resets, 59.2% of conversion to a non-robotic approach, and 81.8% of case abortions [37]. In robotic-assisted knee arthroplasty, device malfunction was responsible for approximately 72% of adverse events. Mechanical failure was responsible for most of the malfunction cases (40.88%), while software failure also played a significant role (31.11%). These issues were noted to prolong surgical time, lead to improper bone resection, and conversion to manual surgery. Of note, almost 60.39% of prolonged cases had the total surgical time extended by more than 20 minutes [38]. In total hip arthroplasty, malfunctions such as unexpected robotic arm movements have been reported, leading to patient injuries and surgical complications [39. The calibration and registration of these devices used in robotic TKA and THA is very sensitive and requires careful and precise preoperative planning. Any errors in the calibration of any one aspect, such as registration of bony landmarks, osteotomy execution, or component implantation, can lead to intraoperative system error or termination of the procedure altogether [40,41].

**Figure 2:**
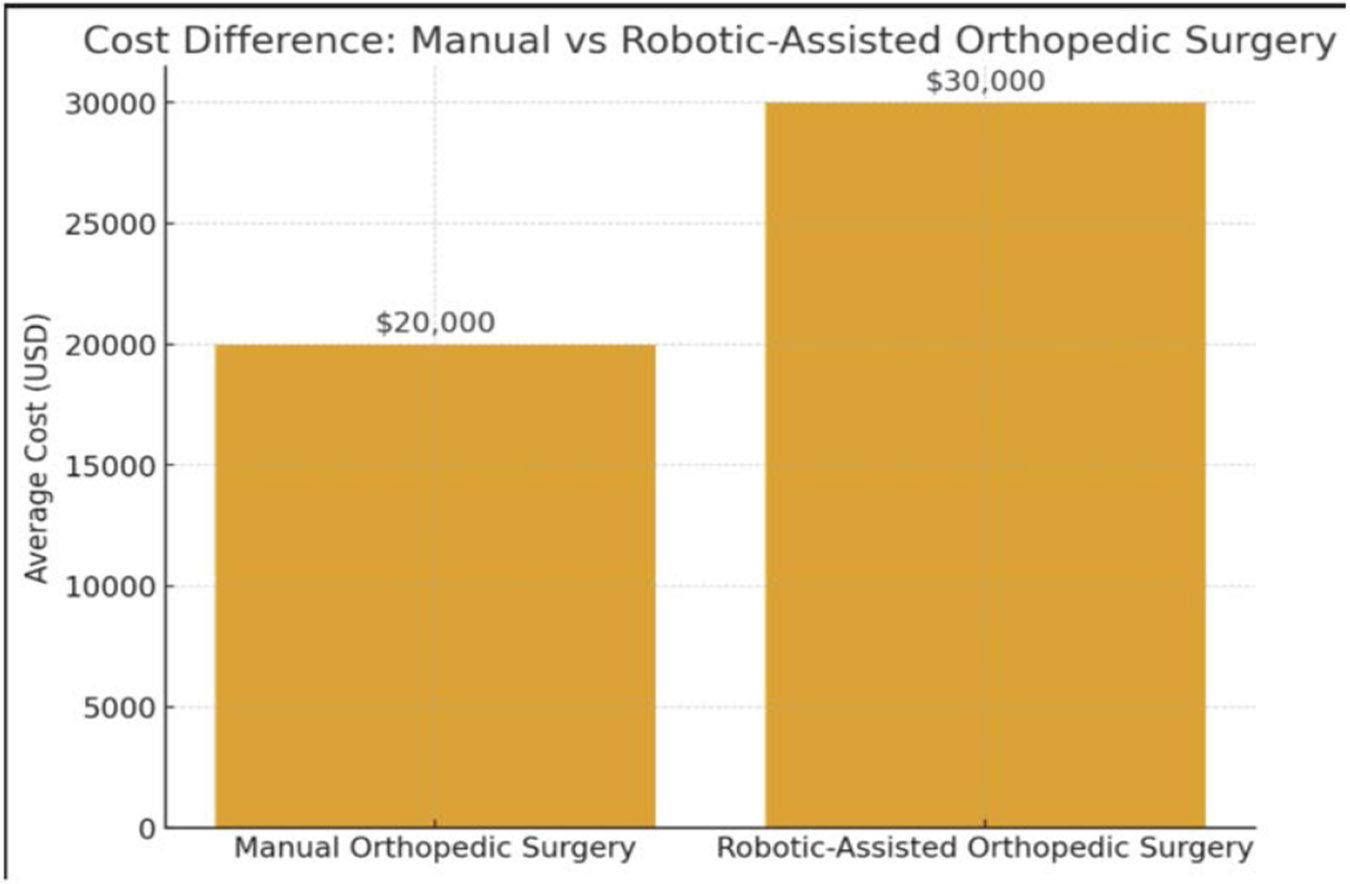
Bar chart comparing the average costs of manual orthopedic surgery (=$20,000) versus robotic-assisted orthopedic surgery (=$30,000). The visualization highlights the increased financial burden associated with robotic systems, which is an important consideration alongside their potential benefits in precision and patient outcomes.

The implementation of robotic-assistance in surgery is also limited by financial and cost-related barriers. Robotic systems require substantial investment from the hospital, including initial purchasing costs and ongoing maintenance costs [42,43]. The average acquisition cost for a robotic system in total knee arthroplasty is approximately US $600,000 to US $700,000, with maintenance contracts typically increasing the upfront cost for healthcare centers [44,45] (Figure 2). The cost-effectiveness of robotic-assisted procedures is highly dependent on hospital procedure volume. Robotic procedures tend to incur higher charges for the surgical procedure itself, but reduced length of stay, fewer complications, and lower rates of post-acute facility discharge drive down risk-adjusted 90-day episode spending at higher volumes [46,47]. A Markov model determined that per case costs for robotic assisted TKA differed by hospital volume: $71,025 (low, 10 cases), $7,463 (mid, 100 cases), and $3,931 (high, 200 cases). The average number needed to treat for cost-effectiveness in this model was determined to be at least 42 cases per year [46]. A separate analytic model confirmed similar findings, with robotic TKA only crossing the cost-effectiveness threshold in 50.4% of cases, often requiring a hospital volume of 49 robotic-assisted TKA procedures to be considered favorable [47].

In the early adoption period, there was limited availability of robotic-assisted procedures, and the lack of evidence regarding benefits restricted the coverage of these technologies in orthopedics [48,49]. However, as their safety and efficacy has become established, reimbursement rates have been documented to be similar for conventional and robotic-assisted cases at least in the cases of many genitourinary and gynecologic procedures [50]. Further research is required to determine if similar patterns have been adopted in the reimbursement of orthopedic robotic procedures.

Implementation is also limited based on surgeon training requirements and steep learning curve. This initial adoption phase is associated with significantly longer operative times. In TKA, the learning curve ranges from 9 to 43 cases, with surgeons with prior arthroplasty experience demonstrating quicker adaptability [51-54]. On average, operative time increases from 12 to 22 minutes per case [47,48-51]. Case aspects with the greatest contribution to increased operative times include device-specific navigation registration and bone resection [55]. However, despite physician inexperience and longer surgical times during the initial learning period, implant positioning accuracy, alignment, and complications rates remained similar [55,56].

There are several clinical limitations to consider. Given that most surgical robots operate based on pre-programmed parameters, they lack the intraoperative adaptability to make real-time adjustments like a trained orthopedic surgeon [56]. In addition, robotic systems often have limited tactile feedback compared to manual surgery procedures. Many models have developed haptic feedback systems to address this limitation. In animal models, procedures performed with tactile feedback had significantly lower grasping forces and decreased rates of tissue damage compared to use of the robotic system without vibration feedback [57]. A meta-analysis of 56 studies demonstrated that robotic systems with haptic feedback were significantly more effective at reducing intraoperative forces, shortening task completion, and improving accuracy [58]. Surgeons prefer these feedback systems, with reported greater awareness of surgical tool interactions, with no interference with their perception of the procedure or use of the robot [59].

Given that robotic systems have only recently gained popularity, there is limited research regarding long-term outcomes for patients undergoing robotic versus manual procedures. Selection bias is a significant concern. Most studies enroll selected populations with limited complications and complexity, limiting generalizability to the broader population. Additionally, many trials are conducted in high-volume centers with experienced surgeons, which may not reflect outcomes in other clinical practice settings.

## Ethical and Practical Considerations of Robotic Surgery

While robotic-assisted surgery in orthopedics has rapidly evolved, offering enhanced precision and reproducibility in procedures such as joint arthroplasty, the ethical landscape is complex, particularly regarding informed consent and patient expectations [60, 61]. Some have suggested that patients may have misconceptions that overestimate the benefits of robotic systems, believing they lead to superior outcomes and fewer complications, despite limited evidence supporting these perceptions [62]. This underscores the ethical obligation surgeons must provide clear, accurate information about the capabilities and limitations of robotic systems during the consent process, ensuring patient autonomy is respected [61,62].

Another significant consideration with the use of robotics in orthopedic surgery is the balance between the surgeon's autonomy in conducting the procedure versus the surgeon’s dependence on the machine. While robotic systems have the potential to improve surgical accuracy, they may also risk diminishing the surgeon's role in intraoperative decision-making, especially as systems become more automatic and require increasingly less human input [63]. Some argue that, regardless of the type of robotic system, the surgeon remains responsible for operative planning and oversight, which is the most impactful aspect of the procedure, while robots simply execute predefined surgical directives [60,61]. A surgeon’s clinical and anatomical knowledge is still what guides the surgery, while the robot helps maximize technical precision and accuracy. Nevertheless, concerns persist about the potential for over-reliance on technology, which could erode clinical judgement and accountability, particularly in the event of adverse outcomes [64]. Ethical frameworks must balance technological advancement with the irreplaceable value of human expertise and judgement. Therefore, with increasing use of robotic devices in orthopedic surgery, additional training and regulations are required to ensure surgeons can reap the benefits of these technologies without allowing these technologies to substitute clinical judgement.

One-way surgeons can use these technologies without being over-reliant is through robust credentialing and ongoing proficiency assessments. The literature highlights that current credentialing requirements are highly variable and often insufficient, with most institutions relying on a minimal number of proctored cases and lacking objective performance assessments or outcome monitoring [1]. The recommendation is to implement standardized, rigorous credentialing guidelines that include not only initial training but also continuous evaluation of technical skills and complication management, as well as regular case volume requirements to maintain privileges. Another suggestion is having structured training programs that involve simulation-based modules, supervised cases, and explicit instruction on recognizing and managing device failures and intraoperative complications. The literature stresses that robotic platforms are shared-control systems, and surgeons must retain primary control and be adept at troubleshooting technical pitfalls to avoid over-reliance on the device. This approach supports ethical informed consent by ensuring surgeons can accurately understand and communicate risks to patients, as well as being actively prepared for possible surgical complications.

Furthermore, disparities in access between high- and low-resource settings present significant ethical and practical challenges. The high initial investment and ongoing costs associated with robotic systems limit their availability to well-resourced institutions, exacerbating inequities in surgical care. The literature calls for strategies to bridge the digital divide, including global collaboration and adaptable regulations, to ensure equitable access to the benefits of robotic-assisted surgery.

To address these disparities, regulatory bodies and healthcare systems should promote equitable distribution of robotic technology and training resources. Strategies include centralizing robotic services in regional centers, subsidizing training for surgeons in low-resource settings, and fostering collaborative networks for knowledge sharing. The literature identifies high costs and proprietary technology as barriers and even recommends public policy interventions to mitigate these inequities. Addressing these disparities is essential to uphold the principle of justice in orthopedic care.

Finally, the integration of robotics in orthopedics raises broader ethical issues, including data privacy, algorithmic bias, and transparency in decision-making. Robust data handling practices and ongoing monitoring are necessary to safeguard patient privacy and ensure fairness, particularly as AI-driven systems become more prevalent [61-64]. The development of clear responsibility frameworks and regularly updated ethical guidelines is recommended to guide decision-making and maintain patient trust in this rapidly evolving field.

Overall, while robotic-assisted orthopedic surgery offers substantial promise, its ethical integration requires careful attention to informed consent, surgeon autonomy, and equitable access, which can be supported by robust ethical frameworks, rigorous training programs, and both national and global policy interventions.

## Future Directions in Robotics

### Integration of Artificial Intelligence, Machine Learning, and Augmented Reality

With the increasing usage and acceptance of artificial intelligence (AI), machine learning (ML), and augmented reality (AR) in all fields of medicine, it is imperative to discuss its potential implications within robotic-assisted orthopedic surgery. Within recent years, the convergence of AI, ML, and AR has been rapidly transforming robotic-assisted orthopedic surgery. In various fields, as well as orthopedics, AI and ML algorithms now support diagnostic imaging, risk stratification, and intraoperative decision-making, often matching or exceeding expert clinician performance in tasks such as fracture detection and outcome prediction. Augmented reality platforms are being increasingly utilized for surgical planning, technical education, and intraoperative navigation, offering enhanced visualization and precision without reliance on preoperative advanced imaging. This has both the effect of improving efficiency while still maintaining and even improving precision. With the integration of AI and ML, novel technologies are expected to enable more adaptive, context-aware robotic systems, facilitating personalized surgical workflows and continuous patient monitoring.

Despite these advances, challenges remain in data standardization, algorithmic transparency, and integration of multimodal data streams. The literature stresses a need for robust validation and clinician oversight to ensure safety and efficacy as AI-driven robotics become more autonomous. Technical solutions such as federated learning and edge computing are being developed to address privacy and data integration concerns, while AR and extended reality tools are anticipated to further optimize surgical training and execution. In addition, continued human oversight will be necessary to ensure these systems are operating properly and that crucial details are not missed by the algorithms. Ongoing research is focused on harmonizing these technologies to maximize clinical impact and minimize unintended consequences.

### Expansion to Other Orthopedic Procedures (Trauma, Pediatric Orthopedics, and others):

Robotic-assisted devices are expanding beyond joint arthroplasty into trauma and pediatric orthopedics, driven by advances in AI and ML. In orthopedic trauma, AI systems have demonstrated high accuracy in fracture detection, classification, and outcome prediction, with sensitivities and specificities frequently exceeding 90%. Predictive models for complications and mortality consistently outperform traditional scoring systems, but most studies remain retrospective and lack external or prospective validation. Thus, the literature calls for robust clinical trials that can validate these tools, as well as for multimodal approaches that integrate diverse data sources and transparent algorithms to support clinical decision-making in complex trauma scenarios.

In pediatric orthopedics, unique anatomical and developmental challenges are present, but early applications of robotics and AI in preoperative planning and intraoperative guidance are emerging. These technologies offer the potential to improve diagnostic accuracy, optimize treatment selection, and enhance safety in pediatric populations. It is important that these technologies be able to accommodate the unique anatomy and size differences of pediatric patients. Therefore, rigorous clinical validation and adaptation to pediatric-specific needs are required before widespread adoption. Future development should focus on integrating point-of-care applications and ensuring that algorithms are generalizable across diverse patient populations and variable anatomical presentations.

### Cost Reduction Strategies and Global Accessibility

While robotic-assisted orthopedic devices are promising and offer the potential for improved efficiency and accuracy, they are expensive. High capital and maintenance costs remain significant barriers to the widespread adoption of robotic-assisted orthopedic technologies, particularly in low-resource settings. For some hospitals or clinical practices, the additional benefits might not be clinically significant enough to warrant additional cost burdens. Proposed cost reduction strategies include modular device architectures, cloud-based analytics, and open-source software solutions, which can lower entry costs and facilitate broader dissemination. Regional centers of excellence and industry-academic partnerships are also recommended to share resources, expertise, and training, thereby improving accessibility and equity in care delivery. If these technologies do become more mainstream, it is also likely that the widespread manufacturing and implementation can become more streamlined, which could eventually lead to diminished costs.

Global accessibility is further supported by the development of remote surgery capabilities, telemedicine integration, and AI-driven decision support, which can democratize access to advanced orthopedic care. The literature highlights the importance of regulatory frameworks that incentivize innovation while maintaining safety standards, as well as policy interventions to mitigate disparities in technology distribution. Global health initiatives are imperative as they can ensure the equitable sharing and use of technologies worldwide. Governments worldwide can also provide subsidies to incentivize the application of these devices and help lower the cost of manufacturing. As cost barriers are addressed, the potential for scalable, high-quality orthopedic care in underserved regions will increase.

### Long-Term Registry Data and Randomized Controlled Trials Needed

The promising nature of robotic assisted devices in orthopedics cannot be underscored. However, there is still the need for long-term registry data and randomized controlled trials (RCTs) to establish the safety, efficacy, and real-world impact of robotic-assisted orthopedic devices. Most published studies are small, retrospective, and lack standardized reporting, limiting generalizability and direct comparison. Comprehensive, privacy-protected registries using deep learning pipelines are essential for post-market surveillance, outcome tracking, and continuous model improvement. These registries will facilitate the identification of patient subgroups most likely to benefit and inform evidence-based guidelines for device deployment.

Prospective RCTs are needed to validate clinical benefits, assess cost-effectiveness, and guide future innovation in robotic-assisted orthopedic surgery. Transparent reporting standards and collaborative data sharing are critical to ensure robust evidence generation and support regulatory decision-making. As the field evolves, ongoing monitoring of emerging clinical trials and registry data will be essential to refine best practices and optimize patient outcomes. As these technologies become more mainstream, continued clinical trials will also be necessary to confirm the safety of these technologies, as well as assess for any long-term post-surgical implications.

## Conclusion

Robotic-assisted orthopedic surgery represents a meaningful advance in surgical precision and reproducibility, with clear advantages in implant positioning, alignment, and improvement in technical consistency. These technologies have demonstrated consistent radiographic improvements and modest short-term clinical benefits, including reduced pain, improved early range of motion, and fewer early complications in certain settings. At the same time, robotic platforms allow for the option for individualized surgical workflows and improved operating room efficiency.

However, these benefits must be weighed against current limitations. The lack of studies reporting long-term patient-reported outcomes, implant survivorship, or revision rates should make surgeons cautious regarding the adoption of robotic devices in orthopedics. In addition, high costs, technical malfunctions, steep learning curves, and reduced surgeon autonomy remain significant concerns. Moreover, variability in outcomes across different robotic platforms and healthcare environments highlights that the impact of these technologies is not uniform. Individual platforms should be tested to confirm those that provide the most consistent satisfactory outcomes. Ethical concerns such as equitable access, informed consent, and over-reliance on technology also require careful consideration as robotics becomes more integrated into orthopedic practice.

Taken together, robotic-assisted surgery should be viewed as a valuable adjunct to surgeons rather than a universal solution. Its adoption should be individualized, guided by patient-specific factors, surgeon expertise, and institutional resources, with decisions anchored in evidence-based practice rather than marketing or patient perception alone. Moreover, knowledge of anatomical structures and choice of surgical approach should still be heavily up to the surgeon, with the robotic device simply aiding in the execution of fine surgical precision and detail. To establish the true clinical value of these systems, high-quality randomized trials, standardized outcome reporting, and long-term registry data are warranted. Only through rigorous and transparent research can the field determine whether the technical advantages of robotic assistance ultimately translate into durable improvements in patient outcomes and justify the substantial costs of its integration.
